# The dataset of Japanese patents and patents’ holding firms in green vehicle powertrains field

**DOI:** 10.1016/j.dib.2022.108524

**Published:** 2022-08-08

**Authors:** Jiaming Jiang, Kensuke Baba, Yu Zhao, Junshi Feng, Sou Kumagai

**Affiliations:** a Graduate School of Humanities and Social Science, Okayama University, Japan; b Cyber-Physical Engineering Informatics Research Core, Okayama University, Japan; c School of Management, Department of Management, Tokyo University of Science, Japan; d Department of Electrical and Communication Engineering, Faculty of Engineering, Okayama University, Japan

**Keywords:** Patents, Green innovation, Vehicle powertrain, Hybrid electric vehicle, Battery electric vehicle, Fuel cell electric vehicles, BEV, battery electric vehicle, EPO, European patent office, FCEV, fuel cell electric vehicles, HEV, hybrid electric vehicle, IPC, international patent classification

## Abstract

In 2020, the Government of Japan declared “2050 carbon neutral” and launched a long-term strategy to create a “virtuous cycle of economy and environment”.^1^ Japanese firms possess many technologies that contribute to decarbonization, which is important to expand investment for Green Technology (environmental technology) development. As automobiles are major contributors to greenhouse gas emissions [1], the technological shift towards **vehicle powertrain systems** is an attempt to lower problems like emissions of carbon dioxide, nitrogen oxides [2]. On the other hand, patent data are the most reliable business performance for applied research and development activities when investigating the knowledge domains or the technology evolution (Wand, 1997). Our paper describes a Japanese patents dataset of the vehicle powertrain systems for hybrid electric vehicle (HEV), battery electric vehicle (BEV) and fuel cell electric vehicles (FCEV). In this paper we create a method of bombinating international patent classification (IPC) and keywords to define “green” patents in vehicle powertrains field, using patent data which were applied to Japan Patent Office recorded on EPO's PATSTAT database during 2010∼2019 year. When analyze patents, it is necessary to consider the social situation of each country including language background, we collect patents description documents (abstracts and titles) not only written in English but also in Japanese. Finally, we build a database includes 6025 green patents’ description documents and 266 patents’ holding firms. With which we then identify 3756 HEV patents, 1716 BEV patents, and 553 FCEV patents. Data about patent holding firms is also appended. The full dataset may be useful to researchers who would like to do further search like natural language processing and machine learning on patent description documents, statistical data analysis for empirical economics.

**Specifications Table**

**Table:** 

Subject	Transportation management
Specific subject area	Green patents on vehicle powertrains, identify green patents using patents’ IPC classification and keywords in description document (title and abstract), comparison tables of searching keywords and patents’ holding firms’ names between English and Japanese
Type of data	Excel file, tables and figures in the article.
How the data were acquired	Applied approximate matching with several keywords related to “green” technology to the Patstat 2021 Spring Edition using Python
Data format	Secondary data Primary dataset is EPO's PATSTAT database and it is available from https://www.epo.org/searching-for-patents/business/patstat.html
Description of data collection	Among all patents applied to Japan Patent Office recorded on EPO's PATSTAT database during 2010∼2019 year, we defined 6025 “green” patents and 266 green patents’ holding firms using method of bombinating IPC and keywords. With which we then classified them into 3756 HEV patents,1716 BEV patents, 553 FCEV patents. Finally, we appended information of patents’ holding firms’ financial data such as number of employees, capital, assets in the fiscal year 2021.
Data source location	• Institution:School of Management, Department of Management, Tokyo University of Science • City/Town/Region: Tokyo • Country: Japan • 1–3 Kagurazaka, Shinjuku-ku, Tokyo 162–8601, Japan
Data accessibility	Repository name: Mendeley Data Data identification number: 10.17632/kysvwvcxmf.1 Direct URL to data: https://data.mendeley.com/datasets/kysvwvcxmf/1

**Value of the Data**

• Our dataset has a high level of completeness, it includes documents both in English and Japanese.
• We propose a method of bombinating IPC and keywords to define “green” patents in vehicle powertrain systems.
• Our dataset makes it possible to survey Japanese firms’ financial data and their holding patents simultaneously.
• Our dataset can be used for merging further information or connecting with other databases.
• Our dataset is useful to researchers who would like to do further research like natural language processing and machine learning, statistical analysis.
• Our dataset is meaningful for forecasting development of new technology and encouraging more environmental innovation.

## 1 Data Description

A Japanese patents dataset of the vehicle powertrain systems for HEV, BEV and FCEV. We define “green” patents in vehicle powertrains field, using patent data which were applied to Japan Patent Office recorded on EPO's PATSTAT database during 2010∼2019. We summarize data into several sheets according to their attributions. The first “patent” sheet is the original data includes whole information we collected from PATSTAT. In the 2nd “consolidated accounting” sheet and the 3rd “consolidated accounting” sheet, we surveyed the financial conditions for the 266 “green” patent-holding firms in the fiscal year 2021. We collected patent holding firms’ banking data that are available in their annual securities reports.^2^ In the next sheet we categorized patents holding firms into nine groups using their company sizes. Finally, we make comparison tables of keywords and firms’ names between English and Japanese in the last two sheets of the dataset.

**Fig. 1:**
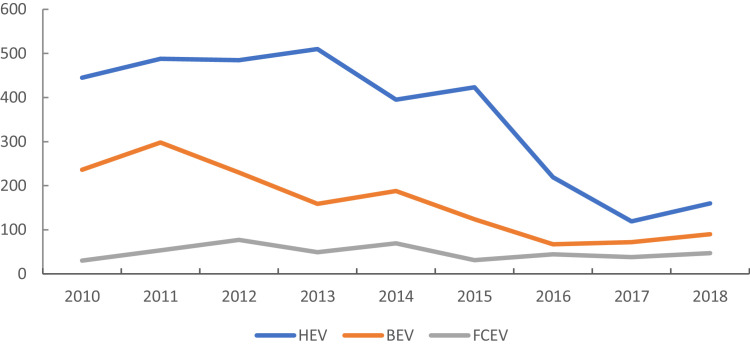
The number of BEV, FCEV, and HEV in 2010–2018.

**Fig. 2:**
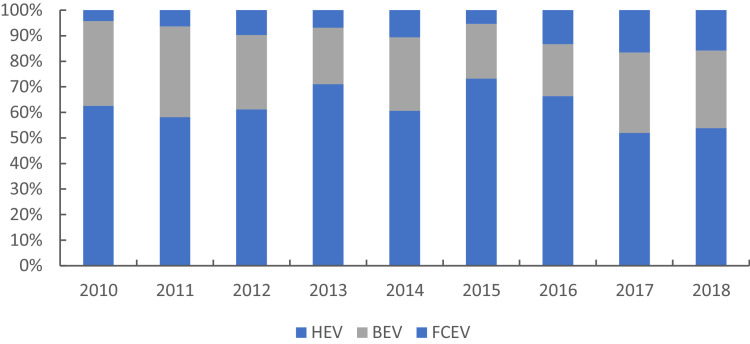
The proportion of HEV, BEV, and FCEV in 2010–2018.

**Fig. 3:**
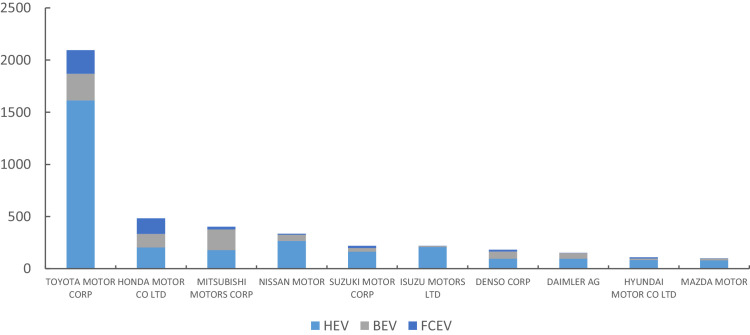
Top 10 firms have large number of green patents (2010–2018).

We make Table 1, Fig. 1, Fig. 2, Fig. 3 base on the 1st “patent” sheet of dataset. As shown in Table 1, our database includes 6025 “green” patents and 266 green patents’ holding firms. With which we identified 3756 HEV patents, 1716 BEV patents, 553 FCEV patents. Then we can observe in Fig. 1, the number of HEV patents increased and peaked in 2013 and showed a downward trend lately, especially was the least in 2017. The trend of BEV is almost the same with HEV but peaked in 2011. Furthermore, the number of FCEV is the least and showed a relatively flat trend.

**Table 1:** The absolute relative and cumulative number of green patents in each powertrain technological field (2010–2019).

	Absolute number			Relative number			Cumulative number		
	HEV	BEV	FCEV	HEV	BEV	FCEV	HEV	BEV	FCEV
2010	445	236	30	63%	33%	4%	445	236	30
2011	488	298	53	58%	36%	6%	933	534	83
2012	485	230	77	61%	29%	10%	1418	764	160
2013	510	159	49	71%	22%	7%	1928	923	209
2014	395	188	69	61%	29%	11%	2323	1111	278
2015	423	124	31	73%	21%	5%	2746	1235	309
2016	380	123	66	67%	22%	12%	3126	1358	375
2017	214	141	78	49%	33%	18%	3340	1499	453
2018	293	167	78	54%	31%	14%	3633	1666	531
2019	85	39	18	60%	27%	13%	3718	1705	549
missing value	38	11	4	72%	21%	8%	3756	1716	553
Total	3756	1716	553	62%	28%	9%	–	–	–

Fig. 2 shows most patents are classified into HEV, less in BEV and the least into FCEV. However, the proportion of BEV and FCEV increased in recent years.

The Fig. 3 shows TOYOTA MOTOR CORP has the largest number of patents, and the most are HEV patents. HONDA MOTOR CO LTD has the second largest number of patents, and has a relatively balanced proportion of HEV, BEV, and FCEV. The proportion of BEV is largest in MITSUBISHI MOTORS CORP. The other top firms have large number of green patents are NISSAN MOTOR, SUZUKI MOTOR CORP, ISUZU MOTORS LTD, DENSO CORP, DAIMLER AG, HYUNDAI MOTOR CO LTD, MAZDA MOTOR.

Table 2 is a list of financial indicators commonly used by patent holding firms in 2nd “consolidated accounting” and 3rd “non-consolidated accounting” sheet of our dataset, which are useful for assessing the performance of innovative activities of patent holding firms.

**Table 2:** List of financial indicators commonly used by patent holding firms.

Description	Variables/indicators
Basic information	doc_std_name (company name) Number of employees (persons)

Cash flow	Cash flows from operating activities (million yen) Cash flows from investing activities (million yen) Cash flows from financing activities (million yen) Free Cash Flow (million yen)

R&D	R&D expense (100 million yen)

Balance sheet	Net assets (million yen) Current assets (million yen) Property, plant and equipment (million yen) Capital expenditure (million yen) Intangible assets (million yen) Investments and other assets (million yen) Non-current assets (million yen) Assets (million yen)

Income statement	Sales (million yen) Cost of Sales (million yen) Gross profit (million yen) Net income (million yen) Selling, general and administrative expenses (million yen) Operating income (million yen) Non-operating income (million yen) Non-operating expenses (million yen) Ordinary profit (million yen)

**Fig. 4:**
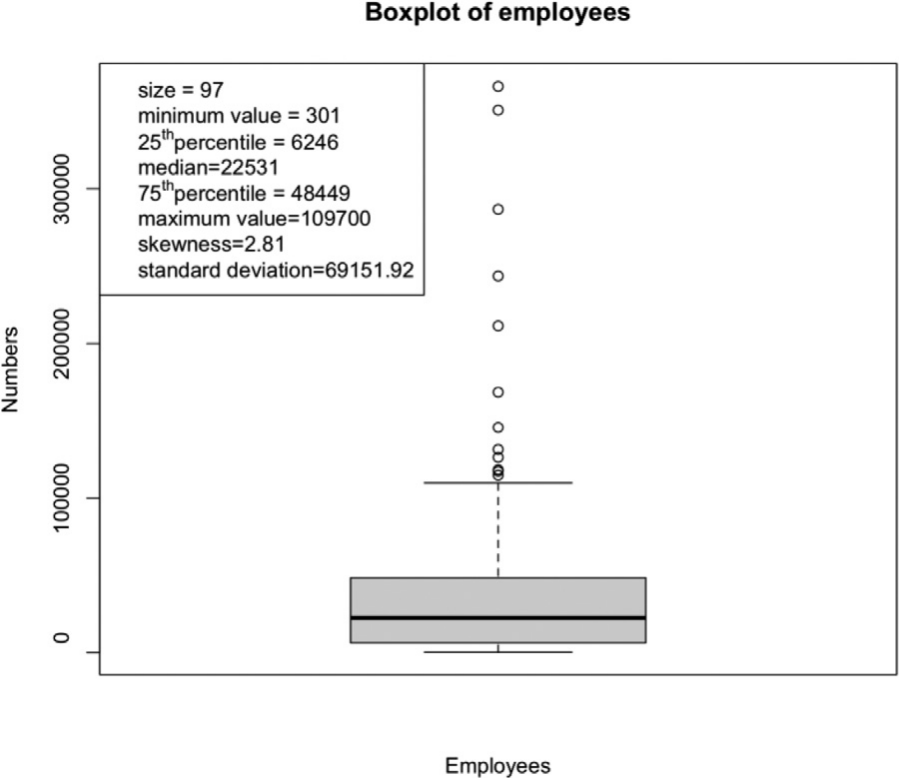
Box-and-whisker plot of the number of employees.

To summarize the financial efforts on the patents, we categorized these 97 firms into nine groups using their company sizes. Fig. 4 shows the box-and-whisker plot of the number of employees. We observe a right-skewed distribution of employees with 12 extreme values, which is due to diversity of these firms. We categorized firms which have extreme values into a big-size group. Furthermore, we computed the bandwidth (10,755) by using the plug-in approach [3,4]. The plug-in approach constructs an estimator of the unknown roughness $R(f^{\left(2\right)})$. A general form of $R(f^{\left(r\right)})$ is

(1) $$\hat{R}\left(f^{\left(r\right)}\right)=n^{-1}\sum_{i=1}^{n}{\hat{f}}^{\left(r\right)}\left(x_{i}\right)=n^{-2}{\tilde{h}}^{\left(-1+r\right)}\sum_{i=1}^{n}\sum_{j=1}^{n}k^{\left(2r\right)}\left(\frac{x_{i}-x_{j}}{\tilde{h}}\right),$$

where $f^{\left(r\right)}$ is defined as the rth derivative of the density function f, and k is the kernel. It is well-known that this estimator depends on a bandwidth and an unknown roughness. To estimate $R(f^{\left(2\right)})$, we need to estimate $R(f^{\left(4\right)})$ to obtain an asymptotically valid bandwidth. The estimation of $R(f^{\left(4\right)})$ further requires a bandwidth $\tilde{h}$, which in turn would depend on $R(f^{\left(6\right)})$. Assuming normality, the roughness of the 6th derivative of a density belonging to the $N(0,\sigma^{2})$ family is estimated by $\hat{R}\left(\phi^{\left(6\right)}\right)=-15/\left(16{\hat{\sigma }}^{7}\sqrt{\pi }\right)$ where  $\hat{\sigma }$ is the estimate of the standard deviation of the random variable x, and $R\left(\phi^{\left(2r\right)}\right)=\left[R\left(f^{\left(6\right)}\right)\left(2r\right)!\right]/\left[{\left(2\sigma \right)}^{2r+1}r!\sqrt{\pi }\right]$. Then the optimal bandwidth for estimating $R(f^{\left(2\right)})$ can be estimated by

(2) $$\tilde{h}={\left[\frac{-2k^{\left(4\right)}\left(0\right)}{\kappa_{2}\left(k\right)\hat{R}\left(\phi^{\left(6\right)}\right)n}\right]}^{\frac{1}{7}},$$

where $\kappa_{2}\left(k\right)$ is the second moment of the kernel. The Eq. (2) coincides with the estimator proposed in [4]. Using $\tilde{h}$, we can estimate the roughness $\hat{R}\left(f^{\left(2\right)}\right)$ via (1). Finally, the optimal bandwidth is constructed by

(3) $$\hat{h}={\left[\frac{R\left(k\right)}{\kappa_{2}^{2}\left(k\right)\hat{R}\left(f^{2}\right)n}\right]}^{\frac{1}{7}}.$$

After excluding all extreme values, we calculated the optimal bandwidth using (3) and the result is 10,755.

**Fig. 5:**
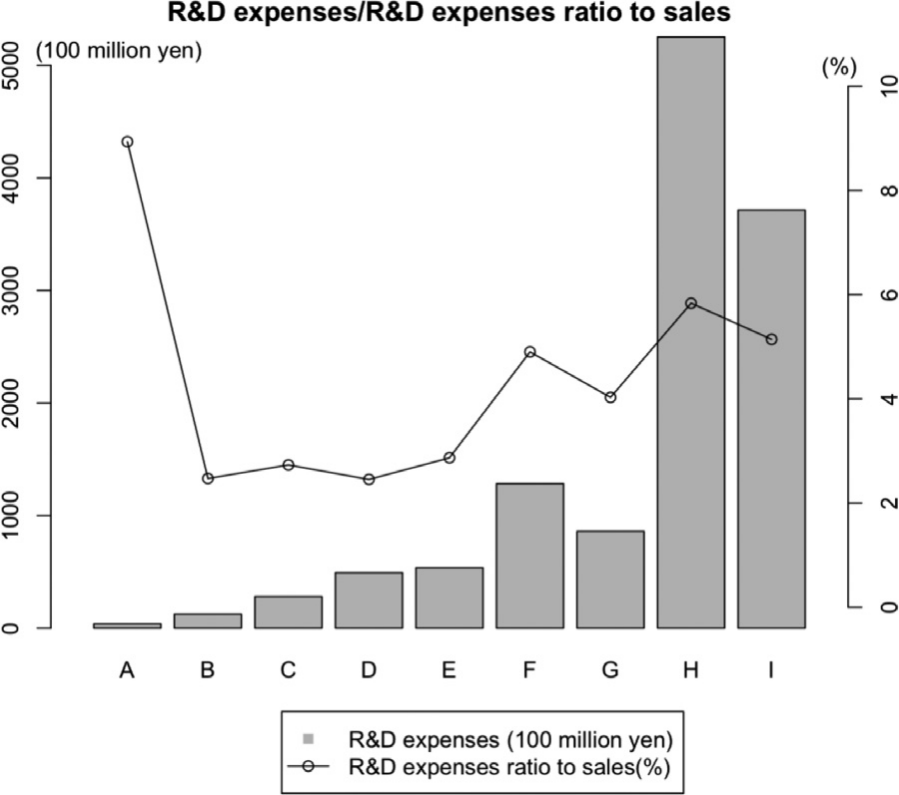
R&D expenses/R&D expenses ratio to sales.

Using the bandwidth, we obtained nine groups labelled by alphabets “A” to “I” in the order of increasing sizes, the details are in the 4th Group (A∼I) sheet of the dataset. The R&D expenses (Billions of yen) and R&D expenses ratio to sales (%) of each group are summarized in Fig. 5. We can find that small-size firms tend to have a higher R&D expenses ratio to sales, considering their levels of R&D expenses.

## 2 Experimental Design, Materials and Methods

### 2.1 Acquire data from Patstat

We acquire data from patstat, which is a worldwide patent statistical database created and maintained by the European Patent Office (EPO)^3^ [5]. Data acquiring methods are in Table 3

**Table 3:** Data acquiring method from patstat.

Attribute	acquiring content	Attribute description
APPLN_ID		Application identification: Technical unique identifier without any business meaning
APPLN_FILING_YEAR	2010∼2019	Year of the application filing date
APPLN_KIND	A (patent)	Specification of the kind of application
APPLN_AUTH	JP	The competent authority, which is the national, international or regional patent office responsible for the processing of the patent application
APPLN_TITLE		Title of application
APPLN_ABSTRACT		Abstract of application
IPC_CLASS_SYMBOL		IPC symbol (IPC 8th edition)
DOC_STD_NAME		Standard name attributed to applicant and inventor names

**Table 4:** Searching strategy.

vehicles_classification	IPC_class	keywords
Hybrid electric vehicles [HEV]	B60K*	(“automobile*” OR “vehicle*” OR “car*”) AND (“hybrid vehicle*” OR “hybrid electric vehicle*” OR “hybrid propulsion” OR “hybridelectric” OR “hybrid car*” OR “plug-in hybrid vehicle” OR “charge-in hybrid vehicle” OR “hybrid automobile*” OR “hybrid electric car*”)
	F02*	
	F16H*	
	B60W*	
	B60L*	

Battery electric vehicles [BEV]	H02K*	(“automobile*” OR “vehicle*” OR “car*”) AND (“electric vehicle*” OR “electric car” OR “electric automobile*”) AND (“battery” or “batteries”)
	H01M*	
	B60L*	
	B60K*	
	B60W*	

Fuel cell electric vehicles [FCEV]	B60W*	(vehicle* OR car OR automobile*) AND ("fuel cell*")
	B60L*	
	H01M*	

### 2.2 Searching strategy with Python

We search patents’ title and abstract acquired last section using IPC classification and keywords. Green patents’ IPC classifications are supplied by IPC GREEN INVENTORY,^4^ keywords are referred to some former researches [2,^6^,7]. We also search patents’ title and abstract written in Japanese, the comparison table of keywords may be found on the final sheet of our dataset.

### 2.3 Append financial data of patents’ holding firms

We collected the financial (banking) data of 97 green patent-holding firms using both the consolidated and non-consolidated accounting in the fiscal year 2021. These data can be effectively used to measure the performance of patent-holding firms and provide useful insights into understanding new vehicle powertrain industry. For example, previous studies on efficiency and productivity analysis such as [8,9] analyzed both the consolidated and non-consolidated data in either a parametric (e.g., stochastic frontier analysis [10]) or a nonparametric approach (e.g., data envelopment analysis [11]).

## CRediT authorship contribution statement

**Jiaming Jiang:** Resources, Software, Writing – original draft. **Kensuke Baba:** Conceptualization, Software, Supervision. **Yu Zhao:** Resources, Software, Writing – review & editing. **Junshi Feng:** Data curation. **Sou Kumagai:** Data curation.

## Declaration of Competing Interest

The authors declare that they have no known competing financial interests or personal relationships that could have appeared to influence the work reported in this paper.

The authors declare the following financial interests/personal relationships which may be considered as potential competing interests:

## Data Availability

The dataset of Japanese patents and patents’ holding firms in green vehicle powertrains field (Original data) (Mendeley Data).

## Footnotes

2 The annual securities report of patent-holding firms can be found either on their websites or the electronic disclosure system (EDINET) operated by the Financial Service Agency of Japan (https://disclosure.edinet-fsa.go.jp/).

3 See https://www.epo.org/searching-for-patents/business/patstat.html.

4 See https://www.wipo.int/classifications/ipc/green-inventory/home.
